# Deep Neck Infection and Descending Mediastinitis as a Complication of* Propionibacterium acnes* Odontogenic Infection

**DOI:** 10.1155/2015/190134

**Published:** 2015-11-29

**Authors:** Evgeni Brotfain, Leonid Koyfman, Lisa Saidel-Odes, Abraham Borer, Yael Refaely, Moti Klein

**Affiliations:** ^1^Department of Anesthesiology and Critical Care, General Intensive Care Unit, Soroka Medical Center, Ben-Gurion University of the Negev, Beer Sheva, Israel; ^2^Department of Infectious Disease, Soroka Medical Center, Ben-Gurion University of the Negev, Beer Sheva, Israel; ^3^Department of Thoracic Surgery, Soroka Medical Center, Ben-Gurion University of the Negev, Beer Sheva, Israel

## Abstract

*Propionibacterium acnes* is an anaerobic, Gram-positive bacterium which causes numerous types of infections. Isolated* Propionibacterium acnes* deep neck infections are very rare. We present an interesting case of deep neck infection complicated by descending mediastinitis of isolated* Propionibacterium acnes* infection.

## 1. Introduction

*Propionibacterium acnes* is an anaerobic, Gram-positive bacterium, comprising a large part of the skin flora of healthy individuals [1].* Propionibacterium* can cause numerous types of infections, including skin [2], corneal [2], dental (periodontitis, periodontal abscess, etc.) [2], orthopedic (septic arthritis) [3, 4], and also heart valve (endocarditis) infections [3, 4]. Deep neck infections caused by* Propionibacterium* are very rare [5]. They are generally polymicrobial (*Propionibacterium acnes* and other anaerobic bacteria, such as* Prevotella* or* Porphyromonas*, and staphylococci or streptococci group) and also have been associated with foreign bodies [5, 6]. Descending mediastinitis is a serious, life-threatening, and usually polymicrobial aerobic and anaerobic infectious complication of common odontogenic and deep neck infections [7]. Even with appropriate management (antibacterial therapy, surgical drainage) the mortality remains higher than 20% [8].

We present interesting case of deep neck infection and descending mediastinitis complicating an isolated* Propionibacterium acnes* odontogenic infection successfully treated in our General Intensive Care Unit (GICU).

## 2. Case Report

**Figure 1:**
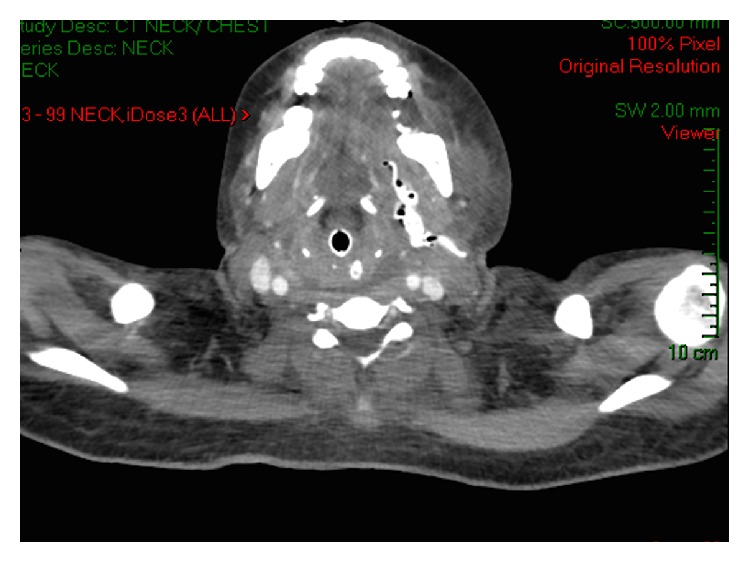
Neck abscesses.

A 21-year-old female, previously healthy, was admitted to the ear-nose-throat (ENT) ward with severe left submandibular nonfluctuant swelling, being erythematous and warm to palpation. Furthermore, mild trismus and sublingual edema were noted. Otherwise, physical and neurological examination was unremarkable. She had a sinus tachycardia of 130 b/min, fever 39°C, and blood pressure 150/90. The chest X-ray on admission was normal. She had a one-week history of a left mandibular second and third molar teeth untreated infection (periodontitis). The patient was urgently transferred to the operating room (OR) where she underwent surgical drainage of the abscess and thereafter admitted to our GICU while sedated, mechanically ventilated, hemodynamically stable, and febrile (38.7°C). Laboratory tests were noted for extensive leukocytosis (30000 cells∖ul). Immediately on ICU admission broad-spectrum antibiotic therapy (ciprofloxacin 400 mg bid and clindamycin 900 mg tid) was initiated. On the following day, extensive bilateral abscesses in parapharyngeal, retropharyngeal, and paratracheal spaces were shown on an urgently performed CT (Figure 1) and the patient was transferred to the OR again for exploration of deep neck infection.

**Figure 2:**
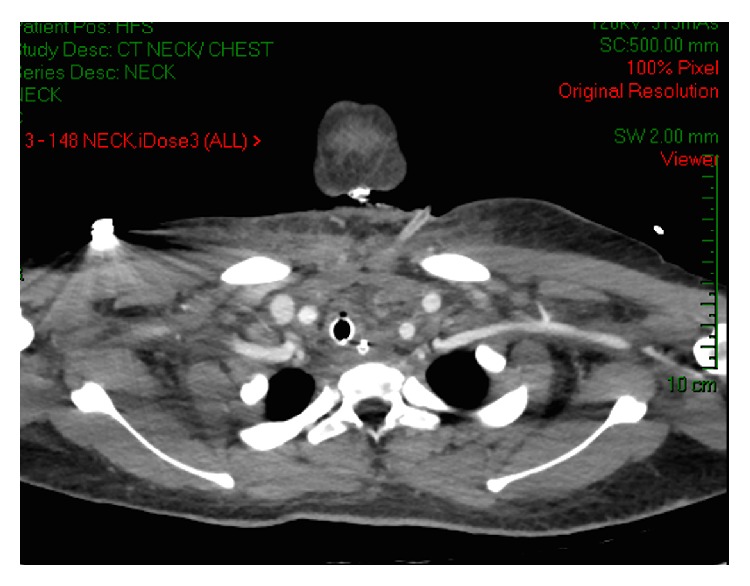
Mediastinal abscesses.

A large amount of purulent fluid was drained; three days later cultures were positive for* Propionibacterium acnes*. The patient remained febrile (39.3°C, WBC of 24.5) though hemodynamically stable. An additional CT scan of the head, neck, and chest with contrast iodine two days later (Figure 2) showed a new abscess in the anterior superior mediastinal space. An urgent right thoracotomy was performed.

A large amount (about 30 cc) of purulent fluid, pH 7.18, was drained from anterior mediastinum and subcarinal space; the patient was transferred back to the GICU. Bacterial culture samples of serous fluid were positive again for* Propionibacterium acnes*. The patient continued antibacterial therapy by clindamycin only due to bacterial susceptibility and isolated growth of* Propionibacterium*. During the next four days she went to OR two additional times for surgical debridement, drainage, and washout of neck wounds. The patient continued the neck wound washout and antibiotic therapy over the next three weeks. She was discharged from the unit four weeks after admission.

## 3. Discussion

Deep neck infections from odontogenic origin are usually a multiple space process [9]. It may be explained by the delay in clinical presentation, which allows the infection to continue spreading along the cervical soft tissue [10]. Mandibular molars are most often the cause of odontogenic infections [11]. Whenever untreated, the infection will spread downwards into the mediastinum and cause a descending mediastinitis, a serious and fatal complication [12]. Both neck and mediastinum infectious processes include mixed aerobic (Gram-positive cocci, commonly streptococci) and anaerobic (*Bacteroides* spp.,* Prevotella*, and* Peptostreptococcus* spp.) pathogens in up to 80% of cases [11, 13, 14].

Our data correlated well with previously published literature. In our case the patient had a preexisting history of untreated teeth infection. This may explain a rapid and down dissemination of the infectious process which involved a large part of neck and anterior mediastinum tissue. The primary and single pathogen isolated in both neck and mediastinum samples of our patient was* Propionibacterium acnes*.* Propionibacterium acnes* is a very rare cause of mediastinitis and deep neck infection [11, 13, 14]. Farmahan et al. (41 patients) [11] and Boscolo-Rizzo et al. [12] found isolation of a single* Propionibacterium* growth only in two cases of deep neck infection. Diamantis et al. [7] described an interesting case of odontogenic infection complicated by descending necrotizing mediastinitis. In that case the infectious process was caused by polymicrobial flora (*Streptococcus constellatus* and* Propionibacterium acnes*) [7]. Boyanova et al. [15] reported two cases of* Propionibacterium* growth (of 118 patients) in deep space head and neck infections. Both of those cases were associated with another anaerobic (*Clostridium tertium* and* Prevotella corporis*) pathogen. There was no growth of other microbial pathogens in the bacterial cultures from our patient. Tammelin et al. described 32 cases of postsurgical mediastinitis after cardiac bypass grafting (CABG) [16]. At least in 25% of mediastinitis* P. acnes* was the primary pathogen [16].

Delay in diagnosis of mediastinitis is believed to be the most significant risk factor for patients' mortality [17]. In the present case we had a high index of suspicion of a disseminating process; therefore repeated CT scans were conducted, demonstrating the spreading of an inflammatory process into the mediastinum space three days after her hospital admission.

An appropriate management of deep neck infection and mediastinitis includes intravenous antibacterial therapy and surgical drainage of the cervical and mediastinal collections [14]. In our case immediate, extensive, and recurrent surgical drainage allowed for a successful and early control of the source of infection.

## 4. Conclusion

Unrecognized and untreated odontogenic infection might be complicated by fatal and life-threatening deep neck infection and mediastinitis. A commonly known pathogen of dental infection,* Propionibacterium acnes*, might be isolated as a single microbial agent of extended deep neck abscesses and even descending necrotizing mediastinitis.
